# Application of Antimicrobial Peptides on Biomedical Implants: Three Ways to Pursue Peptide Coatings

**DOI:** 10.3390/ijms222413212

**Published:** 2021-12-08

**Authors:** Marco G. Drexelius, Ines Neundorf

**Affiliations:** Institute for Biochemistry, Department of Chemistry, Faculty of Mathematics and Natural Sciences, University of Cologne, Zuelpicher Str. 47a, 50674 Cologne, Germany; marco.drexelius@uni-koeln.de

**Keywords:** antimicrobial peptides, surface coating, therapeutic potential, biomaterials, peptide synthesis

## Abstract

Biofilm formation and inflammations are number one reasons of implant failure and cause a severe number of postoperative complications every year. To functionalize implant surfaces with antibiotic agents provides perspectives to minimize and/or prevent bacterial adhesion and proliferation. In recent years, antimicrobial peptides (AMP) have been evolved as promising alternatives to commonly used antibiotics, and have been seen as potent candidates for antimicrobial surface coatings. This review aims to summarize recent developments in this field and to highlight examples of the most common techniques used for preparing such AMP-based medical devices. We will report on three different ways to pursue peptide coatings, using either binding sequences (primary approach), linker layers (secondary approach), or loading in matrixes which offer a defined release (tertiary approach). All of them will be discussed in the light of current research in this area.

## 1. Introduction

The use of medical devices has become part of our daily lives, and within the last years significant progress has been made in the development of such smart and innovative materials. Despite their ongoing improvement main challenges in their application represent implant-associated bacterial wound infections. These cause severe postoperative complications that may even end up in human death, although the surgery process itself was successful. The reason for this is called biofouling, which describes a process when organisms accumulate and colonize on surfaces, which induces infections. Biofilms contain a very heterogenous cell population including metabolically inactive organisms and drug tolerant persisters that survive in the presence of antibiotics [1]. One example of such often occurring incidences displays peri-implantitis being frequently related with dental implants [2,3,4]. However, a high number of implants is placed yearly, and in fact, more than 3 million implants are placed in the USA alone, with a worldwide market growing up to 500,000 implants each year [5]. Notably, other types of medical devices face the same problems, and for instance, biofilm formation on contact lenses may result in various severe ocular diseases such as microbial keratitis [6]. In light of this, it is of upmost importance to find efficient strategies able to prevent infections and thus, to protect the patient [7]. Importantly, the developed solutions should be in any case biocompatible, nontoxic, and environmentally friendly for being useful in health care systems.

One promising strategy in this field is the use of antimicrobial surface coatings on implants or other medical devices to prevent biofilm formation on the material-tissue interface causing such inflammations [8]. Thereby, the risk of bacterial colonization might be decreased, while the overall success of e.g., implant surgery is increased [9]. During the last years, different types of coatings have been developed and tested, including layers of metals that exhibit antimicrobial properties (e.g., silver ions) [10,11], or direct immobilization of antibiotic drugs onto the surface [12]. Herein, the group of antimicrobial peptides (AMPs) has gained more and more attention as potent antibiotic surface coatings [13]. These peptides combine a broad spectrum of antimicrobial activity against a wide range of pathogens with a rather decent cytotoxicity against mammalian host cells [14]. Moreover, most AMPs facilitate a mechanism of action, which is usually not that easily adaptable by bacteria: they display high membrane-activity and lyse the bacterial outer membrane by membrane disruption [15]. Although exceptions exist that kill bacteria also through different mechanism, it is in most cases observed that bacteria treated with AMPs show damaged cell membranes [16]. The fact that membrane disruption displays an alternative mechanism that is opposite to commonly used antibacterial drugs makes AMPs interesting within the field of antimicrobial research, and particularly in spite of innovative new implant coatings [17].

**Figure 1:**
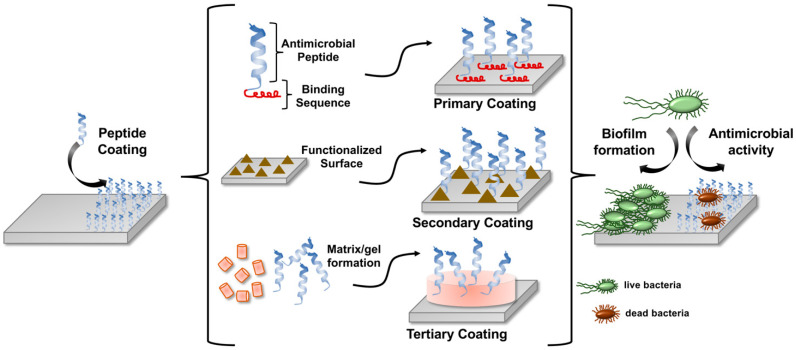
Coating medical devices with antimicrobial peptides is a useful strategy to prevent the formation of biofilms. In a first step, the implant surface is decorated with a layer of AMPs by different means. In this work, we will discuss three different strategies, primary, secondary, and tertiary coating. All of them lead to biofunctionalized solid materials that prevent the colonization with bacteria.

However, one major challenge in establishing antimicrobial peptides as coating materials is their efficient and safe immobilization onto the metal surface. Often such coatings demand harsh chemical conditions, which can lead to limited cell integration on the implants. During the past years, the intense development within this field has often concentrated on three different coating strategies, which are exemplified in Figure 1. First, in a way of primary coating, the peptide sequences are directly coated onto the substrates, e.g., by incorporating metal-binding peptide sequences [18,19]. Secondary coating is meant when in a first step a layer is applied that will deliver a functional group to couple peptides covalently via those linkers onto the substrate [9,20]. Within a tertiary coating strategy, the peptides are embedded into a matrix layer or scaffold and are then released over time [21]. This latter approach differs from the other two in that the peptides are first encapsulated within this hydrogel or matrix, which is then applied as layer on the material surface. Therefore, they are most probably not in direct physical contact with the substrate.

Besides minimizing the risk of bacterial infection, another aspect is that surface functionalization helps to improve the safety of the solid material for clinical applications. Although many materials show great promise in biomedicine, concerns over their biocompatibility have prevented their use in clinical applications. This is partly due to their instability in physiological conditions leading to e.g., corrosion and toxicity in vivo [22]. For instance, stainless-steel is the least corrosion resistant, and metal ions may slowly diffuse through the oxide layer and accumulate in tissue. Thus, it is employed for temporary use only. On the other side, cobalt chrome or titanium alloys do not corrode in the body, and are therefore widely used for the manufacturing of medical devices. Both stand out for their inherent mechanical strength, well-tolerability and biocompatibility [23]. However, owing to its highly frequent use in combination with AMP coatings, titanium will be one of the main highlighted metallic materials within this review. Nevertheless, other materials are also quite popular in the field of dentistry, orthopedics, or plastic surgery including for instance ceramic composites and scaffolds. Those display favorable osteoconductive properties and are, therefore, often used for bone tissue engineering. On the other side, based on the high density and slow biodegradability of ceramics, their further development and use as medical devices is highly challenging [24]. Another example that has become increasingly popular recently, comprises the polymeric material polyetheretherketone, also known as PEEK, which is characterized by its high thermostability and potential for high-load. Since it is highly bone-friendly and more compatible with diagnostic imaging compared to metal materials it has an important role as new standard biomaterial for a wide range of applications [25]. Although quite promising, this material has to be improved concerning its limited bioinertness and lack of antibacterial properties.

## 2. Primary Coating—Use of Specific Metal Binding Sequences

The easiest way of coating a surface with a peptide might be to directly incubate the material with a peptide solution and to dry the peptide on the surface. For instance, this approach was used very recently by Zhang et al., who investigated the effects of antimicrobial peptide Mel4 (KNKRKRRRRRRGGRRRR)-coated titanium plates to prevent postoperative infections in a rabbit model. Since severe infections after internal fixation surgery is an often-seen complication, the antibacterial effect of surface coated titanium plates as alternative treatment option in this field was analyzed. Therefore, rabbits were intravenously inoculated with bacterial suspensions of *Salmonella aureus* or *Pseudomonas aeruginosa* and the effects on Mel4 in treatment of infection after femoral fracture fixation in vivo was measured. The results showed that the AMP-coated titanium plates exhibited great potency against both tested strains, thus offering a promising way to control postoperative infections of open fractures [26]. Of interest was that coating of titanium plates was realized by simply covering the material with peptide solution, following drying under nitrogen. Moreover, the Mel4 peptides were immobilized without having any specific metal binding sequence incorporated. However, within this paragraph the focus will be more on antimicrobial peptides having included a metal binding sequence or other selective groups that induce metal affinity.

Peptide sequences that selectively bind to metal surfaces, such as titanium or stainless steel, were discovered several years ago [22]. They are used to modify the antimicrobial peptide itself giving it the ability to directly attach to the substrate. This strategy offers the advantage to circumvent any adverse effects or interferences that might occur when additional linkers are introduced on the coated material, which might also increase the complexity of the overall immobilization process. In many cases, the respective antimicrobial peptides are directly fused to specific metal binding peptide motives, which were already described as potent to interact with the individual surface properties. However, in some cases, unnatural amino acids or other functional groups might be coupled to the peptide sequence, too, further supporting the adsorption to the surface of the chosen substrates [27]. 

In 2016 Yazici et al. highlighted chimeric peptides composed of a titanium-binding sequence and the antimicrobial peptide E14LKK (LKLLKKLLKLLKKL) [18,28]. Two different metal binding sequences were investigated in this study, namely TiBP1 (RPRENRGRERGL) or TiBP2 (SRPNGYGGSESS), and they were both fused via a simple triple-glycine linker to the AMP. The novel chimeric peptides TiBP1-Spacer-AMP and TiBP2-Spacer-AMP displayed antibacterial activity in solution, wherefore they were then immobilized on titanium plates through simple incubation with the material. Notably, the AMP-coated titanium surfaces significantly reduced bacterial colonialization of *Streptococcus mutans* as well as *Staphylococcus epidermis* and *Escherichia coli* in vitro on the titanium surface [18]. Within this work, the authors demonstrated quite convincing results, however, the underlying mechanisms of activity were not cleared and are content of ongoing studies.

Some years later, Wisdom et al. used this strategy together with the already described binding sequence TiBP1 [19]. The authors aimed to improve dental implants by lowering the risk of inflammation and by making them compatible to surrounding soft and hard tissues. In their work, they evaluated chimeric peptides that comprised three domains: the titanium anchoring domain TiBP (also called TiBP1 [18]), an antimicrobial domain provided by two different AMPs, namely GL13K (GKIIKLKASLKLL) or AMPA (KWKLWKKIEKWGQGIGAVLKWLTTW), and a rigid spacer (GSGGG) to ensure the functionality of the two different domains. By shortly incubating the peptides with the solid material, they were able to achieve nearly complete surface coverage. For their following studies, fluorescently labelled peptides were generated and their surface attachment and stability to serum and mechanical stress was determined. It was found that the AMPA antimicrobial domain had greater helical content and the authors hypothesized that this was the reason that it outperformed the GL13K variant in terms of anti-biofilm formation on *S. mutans* in vitro. Indeed, the authors claimed the TiBP-AMPA peptide as a strong new antimicrobial with high potential as a treatment option for peri-implant diseases [19].

One year earlier, the same group offered a new approach of peptide immobilization providing the possibility to apply peptides on dental implants several times [5]. TiBP-spacer5-AMP (RPRENRGRERGLGSGGGLKLLKKLLKLLKKL) [29] was then repeatedly applied to *S. mutans*-fouled implant surfaces. Therefore, the implants were removed, cleaned with an electronic, round headed toothbrush, and afterwards they were washed with sodium hypochlorite, to remove all bacteria, peptides, and salts. Following, the binding reaction was repeated up to four cycles, and only minimal loss of binding efficacy was detected. In summary, a promising novel non-surgical approach for defouling of bacteria colonized implant surfaces was presented that opens new avenues in the direction of longevity of dental implants [5].

Preventing biofilm formation on titanium was also studied by Zhang et al. in 2018. Herein, several chimeric peptides (TBP-1-hBD3-3, TBP-1-RGDS-hBD3-1, TBP-1-RGDS-hBD3-2 and TBP-1-RGDS-hBD3-3) were built up of three different parts: a linker including an arginine-glycine-aspartate (RGD) motif, the titanium binding peptide TBP-1 (RKLPDAPGMHTW) and one of three different antimicrobial peptides, which were derived from human β-defensin-3, namely hBD3-1 (GINTLQKYYCRVRG), hBD3-2 (GRCAVLSCLPKEQI) and hBD3-3 (GKCSTRGRKCCRRKK). These peptides were coated onto the surface of titanium by incubating them with the material. Antimicrobial activity assays proved their efficiency in reducing *Streptococci* (*S. oralis*, *S. gordonii* and *S. sanguinis*) colonization, assumedly via suppression of sspA and sspB gene expression. Notably, the chimeric peptides did not prevent growing of murine pre-osteoblasts MC3T3-E1 cells on the implant surface demonstrating their high biocompatibility [30]. Based on this work, other TBP-1 containing chimeric peptides were designed, which also included the AMP hBD3-3 and a linker with or without containing the RGD motif. After peptide attachment, activity assays showed that all peptides effectively restricted adhesion and biofilm formation of *S. oralis*, *S. gordonii*, and *S. sanguinis* on the tested implants. This was probably a result of inhibiting the early attachment of bacteria to the surface of the implant. Furthermore, the authors speculated that due to their peptide design of rigid connections charge interactions between the different functional domains had been successfully avoided [31]. This approach might be indeed helpful for the future development of such peptide chimera.

In another work, recombinant silk protein 4RepCT was fused to the antimicrobial peptide Mag-silk (GIGKFLHSAGKFGKAFVGEIMKS) and the cell binding motif FN-silk (CTGRGDSPAC) respectively. Self-assembling proteins may form stable coatings on the surface without covalent attachment so that no additional chemicals are needed, as in the same case when using metal binding peptides. The recombinantly generated silk-AMP proteins were assembled on different surfaces, such as titanium, hydroxyapatite, stainless steel, and polystyrene. High cytocompatibility to human dermal microvascular endothelial cells and human dermal fibroblasts, as well as effective prevention of bacterial adhesion of *S. aureus* was demonstrated. Therefore, those silk protein coatings offer the possibility of future utilization within different orthopaedic and dental implants, coronary stents, or in vitro cell cultures [32].

In a more different approach, Saha et al. embedded the amino acid dopamine (DOPA, **B**) into the peptide sequence, which should act to anchor the peptide onto the material surface. In addition, the fluorinated amino acid 4-fluor-phenylalanine (X) was incorporated (XXXXXKKKKK**B**XXXXXKKKKK) with the idea to create a dual functional coating with both antifouling and antimicrobial activity. In this respect, bacterial adhesion would be reduced and, at the same time, bacteria would be killed that were already deposited on the material surface. Within their peptide design, DOPA should support the adherence to titanium while multiple lysine residues would bring antimicrobial activity and the fluorinated amino acids antibiofouling properties, respectively. Notably, only one DOPA unit in the middle of the 21 amino acid peptide sequence was needed to achieve adhesion to the titanium surface [27]. After immersion of the peptides, activity against *E. coli* and *S. epidermis* was assessed and results showed that the effective concentrations were in the lower micromolar range. Since similar peptides were nontoxic against CHO cells [33], the authors expected their newly designed peptides to be applicable not only for treatments in health care, but also for different industries that would benefit from antifouling metal surfaces like water transport facilities or the food industry [27].

## 3. Secondary Coating—Two Step Approach Including Linker Layers

One other strategy of peptide immobilization onto solid surfaces is to coat the substrates first with an easy to apply layer offering distinct functional groups for the covalent coupling of the peptides. This process is a two-step-approach, and therefore, somehow more time-consuming and laborious than primary coating. The linker layers are quite diverse and originate from organic polymers to inorganic layers, as discussed in the following examples.

One commonly used and highly versatile strategy is to coat the surface with a polydopamine (PD) layer [34]. Here, under basic conditions (using buffers with basic pH) ad-layer functionalization with amine-containing biomolecules, such as peptides or proteins, is possible. For instance, Tan et al. used this conventional way of secondary coating when they tested the bactericidal efficacy of AMP-coated Ti surfaces in a rabbit keratitis model [9]. They first passivated the titanium surface with HNO_3_ solution and then immersed it into a dopamine hydrochloride solution. Afterwards, the PD coated Ti substrates were incubated with the AMP SESB2V ([(RGRKVVRR)_2_K]_2_KK) to yield peptide immobilization. After successful in vitro testing, the modified titanium implants were also evaluated in vivo using infected rabbit corneas. In both cases the material demonstrated effective bactericidal activity against *S. aureus* and *P. aeruginosa*, respectively. Indeed, lower incidence and lesser extent of infection on rabbit corneas with AMP-coated Ti implants were detected compared to controls. The reported results give reason to further evaluate the herein studied novel AMP in its function to prevent not only implant-associated corneal infections, but also the further spread of the infection into fulminant endophthalmitis [9]. 

In 2020 Trzcińska et al. have used polydopamine as a linker layer for attaching antimicrobial peptides on titanium substrates. Therefore, Ti alloys were polymerized with PD in presence of air and three different peptides were selected for the coating. These peptides were based on the LL-37 (LLGDFFRKSKEKIGKEFKRIVQRIKDFLRNLVPRTE) derived AMP KR12 (KRIVQRIKDFLR), namely KR12/32 (KIRVQRIKDFLR), KR12-5911 (KRIVRIKFR), and KR12/32-5911 (KIRVRIKFR), and their antimicrobial activities against *E. coli*, *S. aureus*, and *P. aeruginosa* were shown to be significantly higher compared to parent LL-37. Then, after immobilizing the AMPs on the Ti surfaces their long-term stability and release-profiles in body fluids were analyzed and found to be highly promising. Lastly, human osteosarcoma cells were cultivated on the metal plates and beside the ones modified with KR-12/32-5911, the cells attached densely on all other surfaces, they were viable and able to proliferate. Based on their results, the authors claimed the potential of the herein presented antimicrobial metallic surfaces that were modified with KR12, KR12/32, or KR12-5911, respectively, for application as biomedical materials [35]. However, additional strategies to circumvent the observed toxicity of AMPs have to be developed.

Another solution was presented by Zhan et al. who developed a method to tackle the risk of cytotoxicity that may come with the in vivo use of immobilized AMP HHC-36 (KRWWKWWRR) through polydopamine [36]. For this, they introduced a second layer on PD modified titanium substrates that consisted of a temperature-sensitive polymer (poly(*N*-isopropylacrylamide, pNIPAM). On top of the pNIPAM layer, the AMP was covalently attached using a click chemistry approach. The AMP-coated surfaces exhibited excellent antibacterial activity against *S. aureus* and *E. coli* at room temperature. Following the authors tested in vitro cytotoxicity against rat bone marrow mesenchymal stem cells and rabbit red blood cells and performed in vivo experiments in rabbit legs. Notably, the additional pNIPAM layer promoted a temperature-dependent conformational change at body temperature leading to less exposure of the AMPs and higher biocompatibility in vivo [36].

Polydopamine was also used to assist the immobilization of antimicrobial peptide KR12 on PEEK surfaces to improve its osteointegration and antibacterial properties [37]. The authors demonstrated that the material surface was covalently modified with KR12 without compromising its structure or mechanical characteristics. Furthermore, in vitro and in vivo evaluations of the samples indicated excellent cytocompatibility and osteogenic integration, as well as outstanding osteogenesis. Lastly, promising antibacterial activity was detected against methicillin-sensitive *S. aureus* making this material considerable potential for use as an orthopedic implant. 

Another way of secondary coating is to functionalize surfaces with a layer of inorganic compounds, for instance with alkoxysilane molecules. This method stands out by its low-cost and effectiveness and is usually applied to modify materials that are rich in hydroxyl groups, such as titanium and many other metal oxide surfaces. Silanized surfaces can be further modified since the introduction of active groups (e.g., amino or carboxyl groups) is easily achieved. For instance, components like glass and metal oxide surfaces are covered with such an organofunctional monolayer.

In a recent work, Chen et al. silanized titanium discs with hydrolyzed alkynyl-PEG_3_-triethoxysilane. The functionalized titanium substrates were then treated with a pegylated antimicrobial peptide, namely PEG-HHC36 (N3-PEG12-KRWWKWWRR), containing a terminal azide-functionality for performing a click-reaction that would link the peptide to the substrate. The novel AMP-modified material featured high AMP density and inhibited about 90% bacterial growth when incubated with either *E. coli* or *S. aureus*. Moreover, in vivo antibacterial activity against *S. aureus* and minimal cytotoxicity to mouse bone mesenchymal stem cells were observed giving good prospects for future applications [38].

Silanization was also used in another study by Chen et al. to immobilize the antimicrobial peptide GL13K (GKIIKLKASLKLL) onto titanium surfaces [39]. Here, 3-(chloropropyl)-triethoxysilane (CPTES) was used to add the first layer on the substrate. Afterwards, the AMP was immobilized overnight by dipping the material in a mixed solution of GL13K and Na_2_CO_3_. Those modified materials provided indeed immune regulatory properties and altered macrophage response. In fact, this approach seemed to be very promising since various inflammatory and anti-inflammatory effects were reduced [39]. 

Furthermore, in the work of Koidou et al. silanization of titanium offered the first layer to immobilize laminin 332- and ameloblastin-derived peptides, Lam (KKGGGPPFLMLLKGSTRFC) and Ambn (KKKGGGVPIMDFADPQFPT), respectively. Again, CPTES was taken to fabricate monopeptide and bi-peptide coatings, onto which the two peptides were immobilized either alone or simultaneously. The newly created titanium implants were utilized in preventing peri-implantitis, and indeed, the grafted peptides supported the formation of hemidesmosomes by keratinocytes and promoted epithelial attachment around teeth [40].

Another example was presented in 2020 by Fischer et al. who used silanization to bind peptides and prevent peri-implant infection through creating a structural barrier between the soft tissue and the implant surface. Therefore, they silanized the surfaces of titanium plates with (3-chloropropyl)-triethoxysilane and *N*,*N*-diisopropylethylamine. Those discs were thereafter immersed in the peptide solutions of the two peptides GL13K (GKKIKLKASLKLL) and LAMLG3 (KKGGGPPFLMLLKGSTRFC) that should combine antibiofilm activity (when testing *S. gordonii*) with enhanced proliferation of human keratinocytes. Beside those obtained effects, the coatings were additionally highly stable to mechanical and thermochemical stress [41].

Some years earlier, the antimicrobial peptide FK-16 (FKRIVQRIKDFLRNLV) was covalently attached onto silanized titanium substrates. To accomplish the peptide coupling titanium foils were first hydroxylated and thereafter silanized using (3-aminopropyl)triethoxysilane to obtain free amine groups. Following functionalization with a short bifunctional cross linker (e.g., 6-maleimidoheaxanoic acid) the free maleimide group of the linker was able to react with the thiol group of the *C*-terminal cysteine of FK-16Cys (FKRIVQRIKDFLRNLVC). The decorated Ti surfaces possessed broad-spectrum activity against ESKAPE pathogens and displayed potent anti-adhesion and biofilm inhibition capabilities against *S. aureus* and *E. coli* [42].

Two other examples further prove the feasibility of this secondary coating strategy. Chen et al. immobilized daptomycin on titanium alloys to selectively target cell membranes of gram-positive bacteria. Daptomycin belongs to the group of lipopeptides containing a cyclic structure that includes several modified and d-amino acids. It has been isolated from *Streptomyces roseosporus* and exhibits rapid in vitro bactericidal activity against a variety of gram-positive bacteria [43]. The Ti surfaces were silanized and functionalized with a hydrophilic tetra(ethylene glycol) spacer to which daptomycin was coupled via a thioether linkage. Inhibition of colony formation was tested using *S. aureus* and the foils having the antimicrobial peptides attached demonstrated high self-protecting bactericidal properties. The reported strategy described in this work provided another new way for preparing highly versatile bactericidal implants [44]. Additionally, Hoyos-Nogués et al. offered another technique to attach the cell adhesive RGD sequence (RGDS) and the lactoferrin-derived antimicrobial peptide LF1-11 (GRRRRSVQWCA) via a three-step synthesis approach. They started with silanization of the titanium surface and subsequently cross-linked it with *N*-succinimidyl-3-maleimidopropionate followed by covalent peptide attachment. This multifunctional coating proved to be highly potent to prevent bacterial infections of *S. aureus* and *S. sanguinis* and to support cell adhesion, proliferation, and mineralization of human osteoblast-like cells in vitro [45].

Other strategies of secondary coatings including inorganic compounds were presented by Kazemzadeh-Narba et al. who processed a thin layer of micro-porous calcium phosphate (CaP) on titanium implants to use it as a drug carrier enabling loading and local delivery of cationic AMP Tet213 (KRWWKWWRRC), a modified version of HHC36 [46]. After electrolytic deposition onto the surface of the titanium plates they were analyzed with SEM, XRD, and FTIR confirming the CaP layer to be microporous octacalcium phosphate. To this layer the AMP was coupled via its terminal cysteine thiol-function and loading was determined using a luminescence spectrometer technique. Biological assays then supported that coating with these CaP-Tet213 layers might be a potential solution for preventing infections of implants used in orthopaedics. In fact, highly effective reduction of colony forming was achieved when the implants were tested against Gram-positive *S. aureus* and Gram-negative *P. aeruginosa*, while they exhibited no cytotoxic effects against osteoblast cells [46].

More recently in 2019, Yazici et al. have used electrochemically deposited calcium phosphate as the linking layer between nanotubular titanium surfaces and attached peptides. Therefore, they developed a dual functional peptide (cHABP-1-Spacer-AMP) consisting of the hydroxyapatite binding peptide-1 (cHABP1: CMLPHHGAC) and a peptide of antimicrobial activity (tet-127: KRWWKWWRR, also named HHC36 [36]), both combined with a flexible linker (GGG) [47]. Notably, in this work the peptides were self-assembled by the CaP binding sequence on the metal surface and not covalently integrated as in the studies before. However, the chimeric peptides showed high antimicrobial activity in solution against *E. coli* and *S. mutans*, but also reduced bacterial adhesion when covered on the metal surfaces. The latter was discussed to be strongly correlated to the secondary structure of the peptides.

Another approach of secondary coating was presented by Acosta et al. in 2020 with the aim to increase the antimicrobial potential and bioactivity of the AMP-coating [48]. Herein, organic linkers for peptide tethering on titanium surfaces were established, namely elastin-like recombinamers that resemble protein-engineered polymers, mimic the extracellular matrix and thus, offer simulated in vivo environmental conditions [49]. By using click chemistry the AMP GL13K (GKIIKLKASLKLL) was introduced both, in its d- and l- form, to evaluate the higher proteolytic stability of the d-enantiomer in terms of improved bacterial resistance. In fact, the resulting hybrid antibiofilm coating showed excellent cytocompatibility towards primary fibroblasts, while strong activity against the formation of clinically relevant bacterial biofilms was provided, particularly for the d-Form. The herein highlighted multivalent platform opens up auspicious possibilities in the biofabrication of coatings combining the antibiofilm potential of AMPs and the outstanding tunability and biomechanical properties of the elastin like recombinamers [48].

The following examples highlight strategies in which other implant materials than titanium were modified with a layer of AMPs. For example, secondary coating was used in a study by Monteiro et al. when they aimed to treat urinary catheter-associated infections [20]. For their approach a de novo designed AMP was investigated, namely Chain201D (KWIVWRWRFKR), that stood out not only by its excellent antimicrobial activity against *E. coli*, *S. aureus*, *P. aeruginosa*, and *Klebsiella pneumoniae*, but also by its stability under various conditions, such as high temperature, different pH values and salt concentrations. Within this work, the antimicrobial properties of Chain201D when covalently attached to self-assembled monolayers (SAM) were studied. Therefore, a SAM made of 1-mercapto-11-undecyl-tetra(ethylene glycol), EG4-thiol, was firstly introduced on a gold surface. After activation with 1,1′-carbonyldiimidazole the peptides were attached using basic conditions. The modified substrates exhibited high activity to prevent surface binding and adhesion of strains relevant in the context of urinary catheter-associated infections. Indeed, the presented strategy has the advantage of ease of synthesis, since the AMP is immobilized without any further modification and without the need of an introduced spacer [20].

AMP-modification using glass surfaces as substrates was provided in the work of Yasir et al. [50]. Here, the material was first functionalized with a 4-azidobenzoic acid linker that served to attach two different highly potent AMPs, namely Melimine (TLISWIKNKRKQRPRVSRRRRRRGGRRRR) [51] and a shorter version of it called Mel4 (KNKRKRRRRRRGGRRRR), respectively. Bacterial growth of *P. aeruginosa* was successfully inhibited when AMP-coated glass surfaces were investigated. Notably, when the mechanisms behind were analyzed, it turned out that the peptides seemed to act via the same mechanism of action they show in solution, but that immobilization resulted in much slower kinetics [50].

A dual coating approach was established by Townsend et al. in 2017 when they investigated a novel method to improve the material-tissue interface by AMP surface attachment. For this, they used two different peptides that should be layered both in a covalent and non-covalent approach on hydroxyapatite to yield surfaces with a robust long-term AMP loading and possibility to release active AMP also in the tissue surrounding. The covalent peptide cAMP (RRRRRRGALAGRRRRRRGALAG) was coupled via disulphide bonds on the hydroxyapatite substrate providing a long-term antibacterial film. On the other hand, they deposited the electrostatic peptide eAMP (RRRRRRGALAGRRRRRRGALAGEEEEEEE) via incubation to generate a short-term layer of AMPs that might be released in the tissue environment. The peptides maintained a good activity against a broad spectrum of different bacteria (*S. epidermidis*, *S. aureus*, and *P. aeruginosa*). Moreover, it was highlighted that by using this dual-coating strategy colonization of bacteria could be inhibited and cell-growth could be preserved. All in all, those results looked very promising, although the system was not yet tested in in vivo settings [52].

## 4. Tertiary Coating—Loading and Release from Matrices

The third method presented within this review describes so-called tertiary coating. This means a way of indirect functionalization, in which the peptides are not coated on the surface using covalent bonds or linker layers, but in which the peptides are rather put into a material matrix from which they should be released over time. This matrix is often represented by nanotubes or pores of the same element as the surface, but also other layers such as hydrogels are utilized. One of the advantages might be that the antimicrobial effect is not only provided on the contact surface by the attached peptides, but also within the surrounding tissue after peptide release.

The first subtype of tertiary peptide coating describes the creation of nanostructures of the same material onto the surface of the substrate used for peptide loading. A prominent example is the preparation of titanium nanorods onto the main substrate. This is generally accomplished via electrochemical reactions through anodation. The peptides are subsequently loaded into the tubes and can thus be released into the surrounding tissue, after the implant has been inserted into biological conditions. Li et al. used such titanium nanotubes (TNTs) and combined them with the AMP GL13K (GKIIKLKASLKLL) with the aim to develop antimicrobial coatings for dentistry applications. For this, GL13K was immobilized into the nanotubes by a simple three-fold soaking technique. A slow drug release profile was detected leading to potent antimicrobial properties against *Fusobacterium nucleatum* and *Porphyromonas gingivalis* as well as good biocompatibility to mouse preosteoblastic cells making the GL13K-TNTs interesting novel materials for future application as biomedical implants [53].

To prolong the release of peptides from the TNT reservoirs Zhang et al. have developed a further modification strategy. Two films of different sizes were set on the metal surface, whereby the thinner tubes, called nanocaps, were set on top of the underlying broader tubes, called nanoreservoirs. Finally, the TNT layer was loaded with the AMP ponericin G1 (GWKDWAKKAGGWLKKKGPGMAKAALKAAMQ) using vacuum-assisted physisorption. Interestingly, with these dual-diameter nanotubes the antibacterial activity against planktonic *S. aureus* was prolonged, and cytocompatibility to human fetal osteoblastic cells comparative to that of Ti but higher than that of other AMP-loaded films was achieved [54].

Later on, this approach was further developed by including not only titanium-nanotubes, but also titanium-nanopores. Therefore, both types of layers were applied onto the substrate surface by electrochemical deposition and afterwards loaded with the antimicrobial peptide LL37 through lyophilizing the peptide solution for several repetitive cycles. In that way an almost quantifiably release of the peptides from the titanium pores was observed. Furthermore, an improve in antibacterial activity against *S. aureus* and osteogenic induction was determined making the herein prepared material more suitable for preparing drug-device combined implants for bone injury treatment [55].

More sophisticated was the strategy by Phil et al. who investigated the antibiofilm properties of the AMP RRP9W4N (RRPRPRPRPWWWW) when incorporated into mesoporous titania. This system was generated on different substrates by evaporation induced self-assembly of titanium(IV)tetraethoxide as inorganic precursor together with the block copolymer Pluronic 123 serving as a template. Afterwards the pores were loaded with the peptide by immersing the material in an AMP-solution. In fact, it was shown that the released peptides increased osseointegration and exhibited sufficient antibacterial effects when studying the system in a rabbit tibia model directly at the implant healing site [56].

As the unspecific release of peptides from nanorods might lead to unwanted cytotoxic effects Chen et al. have recently presented another, more advanced approach. The authors used titanium-nanotubes loaded with the antimicrobial peptide HHC36 (KRWWKWWRR) and sealed them with a pH-sensitive poly(methacrylic acid) membrane that would release the peptides when the pH value drops below the value of 6 as a result of infectious conditions. Indeed, the matrixes opened directly when infection occurred, and AMPs were effectively released to kill bacteria. Four clinically relevant bacterial strains were incubated and tested with this nanotube material, including *S. aureus*, *E. coli*, *P. aeruginosa*, and methicillin-resistant *S. aureus*. Besides detection of high antimicrobial activity that inhibited bacterial colonization, the novel compounds proved to be biocompatible in vivo and did not exhibit any cytotoxicity against human mesenchymal stem cells, too [21].

Another way of integrating AMPs non-covalently on the substrate surface is to form a permeable shell-like matrix. Different chemical compositions are used, in which the peptides are loaded for later release including different types of inorganic or biological, often protein-based coatings. For example, Shi et al. employed the biomolecule collagen as a drug-loadable scaffold and demonstrated how a multilayer coating on titanium plates was applied to decrease the growth of bacterial strains responsible for peri-implantitis [57]. Therefore, the broad-spectrum AMP Tet213 (KRWWKWWRRC) was coupled to free amines of collagen IV through the bifunctional linker sulfo-SMPB and the resulting construct (AMPCol) was assembled on smooth titanium surfaces using a layer-by layer technique. Within this layer the peptides were only loosely packed, and thus, were released over time to provide their antimicrobial activity against studied Gram-positive aerobe *S. aureus* and Gram-negative anaerobic *P. gingivalis*. Additionally, only low haemolytic effects and no cytotoxicity against HaCaT cells was detected pointing to very promising biocompatibility. Summarizing, the herein presented AMP-coated material displayed high potential to prevent peri-implantitis accompanied by a favorable long-term activity, since biofilm formation was inhibited up to one month [57].

With the aim to limit the disadvantage of high degradation rates of Ca- and Si-based ceramic (CS) coatings, Zhang et al. used a fluorous-cured collagen shell as scaffold around CS nanorods on Ti implants. To achieve this, they first coated the metal substrates by microarc-oxidation with the CS nanorods, and then further modified these rods with fluorous-cured collagen (Col-1) via spin coating to reduce the natural degradation of CS. Afterwards, this collagen scaffold was loaded with the antimicrobial peptide HHC-36 (KRWWKWWRR) to impart antimicrobial activity while promoting cell adhesion. Notably, this multifunctional approach allowed regulation of the rate of nanorod degradation, and also enhanced cytocompatibility of the implant making it a promising tool for future use in coatings for bone regeneration [58].

Different other types of organic polymers as scaffold components were introduced by López et al. in 2020 [59]. Here, coating of thin polymer multilayers composed of chitosan and hyaluronic acid were designed to load and release an antimicrobial peptidomimetic (β-peptide: (ACHC-β^3^hVal-β^3^hLys)_3_) potent against *S. aureus* biofilm formation. The polymeric layer was generated by sequential immersion of the substrate in solutions of chitosan and hyaluronic acid leading finally to a chemical crosslinked polymeric matrix shell. This polymeric layer was then easily loaded with the antimicrobial β-peptide, and the resultant material indeed proved to be versatile to allow attachment of mammalian cells while reducing biofilm formation [59].

A few years earlier, Cheng et al. described an adhesive, osteoconductive, and antimicrobial hydrogel coating for titanium substrates based on gelatin methacryloyl (GelMA). This matrix was additionally modified with catechol motifs enhancing the adhesion to the surface and thus improving coating stability. Again, the AMP HHC-36 was used for drug-loading into this hydrogel, and a controlled peptide release was observed. Moreover, the herein presented hydrogel coating prevented biofilm formation and supported effective osteogenesis, which was further enhanced by the addition of silicate nanoparticles [60].

On the other side, inorganic material was employed by Liu et al. for building up the scaffold layer. Therefore, titanium alloys were coated with a nano-hydroxyapatite layer via dip coating in hydroxyapatite suspension and subsequent burning in a high-temperature vacuum furnace. Within these porous nano-sized structures both the antimicrobial peptide (hBD3-3: GKCSTRGRKCCRRKK [33]) based on human β-defensin 3, as well as the bone morphogenetic protein-2 were adsorbed. In this study, promising antimicrobial activity, as well as successful cell adhesion, cell proliferation, and osteogenic differentiation of hBMSCs were also obtained [61].

Hydroxyapatite was also used by He et al. to modify the surface of micro-structured titanium substrates. They realized this by following a two-step method including deposition of a hydroxyapatite layer using micro-arc oxidation (MAO) and hydrothermal treatment. Following, an additional polydopamine layer was placed around the micro-structured titanium substrates via dip-coating. This scaffold-layer was then further functionalized with LL-37 by immersing the matrix with different peptide concentrations. Thus, different release profiles of the peptides were obtained, and the viability, adhesion, migration, and osteogenic differentiation of MSCs in vitro was confirmed [62].

Volejnikova et al. probed the activity of AMP variants when implemented in a polymethylmethacrylate (PMMA)-based bone cement model [63]. Indeed, this material is one of the most frequently used cement on orthopaedic surgery. It has been already demonstrated that when this material is loaded with conventional antibiotics, e.g., gentamicin or vancomycin, bacterial adhesion and biofilm formation can be effectively prevented. However, within this study different selected analogues of the AMP halictine-2 (HH27: GKWMKLLKKILK and HH39: GKWVKLLKKILK) were loaded with the aim to improve the overall antibiotic activity of the PMMA bone cement, while being exposed to microbes in the surrounding medium. Thus, the bone cement itself acted as matrix from which the peptides were released. After synthesis of AMP- and, as a control, also antibiotic drug-loaded solid beads, they were tested against several bacterial strains usually responsible for biofilm formation (methicillin-resistant *S. aureus*, *S. epidermidis*, *P. aeruginosa*, and *E. coli*). Strikingly, a nearly total decrease in bacterial cell growth, as well as nearly no bacterial adhesion on the AMP loaded PMMA bone cement beads was measured. The authors found promising release kinetics and good stability of the peptides in the medium. Moreover, their results showed that the AMPs exhibited equal activity, but broader specificity compared to the antibiotics making their approach interesting for future application as biomedical materials in orthopaedic surgery [63].

The following Table 1 provides some information on the AMPs presented in this review.

## 5. Conclusions

Biofunctional surface coatings including antimicrobial peptides are considered a promising strategy to prevent the adhesion of bacterial pathogens and thus, to limit perisurgical infections. Many of such biomaterials display high biocompatibility and provide additional bioactivities, e.g., they allow cell colonization and proliferation, which is important for application in tissue regeneration. However, at the interface between tissue and biomaterial the coatings may suffer from poor stability. This leads to unwanted release and suspension of the functionalized material into the surrounding environment accompanied by decreased activity and/or undesired cytotoxic effects. Therefore, the development of novel biofunctionalizations that own optimized antimicrobial activity and ideal biological tolerance is still highly appreciated. Within this work, we aimed to summarize at least some of the recent efforts made in this direction. A focus was set on the use of titanium as solid substrate, since it is widely applied and many examples including AMPs could be collected. Notably, we identified three major strategies by which the peptides were integrated onto the surfaces including physical adsorption or covalent immobilization.

In a primary coating approach, the AMP itself is modified in most cases by attaching a metal binding sequence or, in fewer cases, by coupling other small chemical compounds or even full proteins. Usually, direct covalent bonds are formed between the AMP and the binding moiety and the conjugate is then non-covalently adsorbed onto the surface. On the one hand, this strategy offers an easy to perform coating step, while, on the other hand, one should investigate the influence of the metal binding sequence to the activity of the AMP beforehand to exclude any detrimental effects. Some other shortcomings of this method include the lower stability in biological surroundings possibly leading to harmful side effects. Additionally, it is not really clear how many molecules will be adsorbed onto the surface and execute the antimicrobial effect. However, to get some quick insights into the overall activity of a newly identified AMP, this primary coating might be a versatile and easy to perform strategy.

Compared to this, secondary coating utilizes another chemical linker layer, that is at first deposited onto the substrate and acts as an anchor to the peptides, which are usually covalently attached to these linkers. This strategy allows to incorporate the peptides in a directed fashion, and also often supports a defined structuring that is very important for AMPs to carry out their antimicrobial activity. Furthermore, in this case, the peptides have to be modified with groups that permit covalent binding to the surface. However, compared to the binding sequences used in primary coatings, they should not alter the bioactivity of the peptides. Here, the complication lies in the first step of functionalizing the solid surface enabling efficient biofunctionalization. On the other side, when these coupling modalities are established, safe and biostable materials are the outcome, which is why we think that this approach is certainly worth taking into consideration for in depth biocompatibility studies.

Lastly, we highlighted tertiary coating as another approach in which the antimicrobial peptides are rather implemented into a matrix or gel supplemented onto the solid surface from which they should be released into the surrounding tissue over time to exert their function. Although somehow more laborious and sophisticated in preparation, this method certainly stands out by the fact that peptides are controllable deliberated over time. Thus, the biological effect can be monitored and critical (too high) concentrations of AMPs in the surrounding tissue can be avoided. Similar to this surface coating are other recently presented techniques that do not use metal surfaces and in which the peptides are directly implemented in the coating material, for example chitosan-gels, hydrogels, or other polymeric adhesive materials that are useful as e.g., dental implants [64,65,66].

One major challenge all methods are faced with is the issue of density of coated AMPs on the used substrate. Besides important parameters have to be considered for a successful and effective coating including for instance the length of the peptides, their orientation and flexibility, or spacer molecules connecting the peptides onto the surface. Therefore, the AMP density itself can affect the successful tethering of the molecule to the material surface [67]. In addition, although various biophysical methods exist that at least proof the existence of an AMP layer on the material, the exact determination of how many molecules are attached is still quite complex. It should be, therefore, one of the main future goals to develop suitable methods that offer a clean analysis and reliable comparison of different coatings. 

Moreover, the high potency of AMPs in the laboratory (be it in solution or immobilized on solid surfaces) does not guarantee a systematic use of them in the near future, and in fact, so far, a bacterial-resistant material has not been developed yet. This is mainly due to limited in vivo activity that is caused by various factors such as low bioavailability, serum inhibition, residual toxicity, and proteolytic degradation. Another important aspect is that the conditions tested for implants in vitro greatly differ from that in real life, which are often represented by medium to high salt environments and/or acidic media. For instance, salinity in an eye environment is higher than the one tested in vitro. In fact, many promising AMPs show reduced activity in the presence of physiological concentrations of salts and other biological fluids. For a further improvement all these limitations should be carefully evaluated and novel strategies should be devised to overcome these downsides. One important method for enhancing stability in body fluids might be to introduce modifications in the AMP structure and thus to improve physico-chemical characteristics. Since many AMPs are synthesized chemically, their production including unusual amino acid building blocks should be feasible.

In conclusion, all of these strategies have proven their feasibility and, more importantly, helped to define essential parameters for the further design of such biomaterials that should exhibit high biocompatibility and excellent antimicrobial activity in vitro and in vivo.

## Figures and Tables

**Table 1:** Sequences and selected physicochemical properties of most peptides featured in this work. (* GAH: grand average hydrophobicity; ** X: 4-fluor-phenylalanine; B DOPA).

Name	Sequence	Refs.	*M*_W_	pI	Charge	GAH *
Ambn	KKKGGGVPIMDFADPQFPT	[42]	2033	10	1	−0.6
E14LKK/AMP	LKLLKKLLKLLKKL	[18,28]	1692	11	5	0.5
AMPA	KWKLWKKIEKWGQGIGAVLKWLTTW	[19]	3085	10	5	−0.4
cAMP	RRRRRRGALAGRRRRRRGALAG	[52]	2631	13	11	−1.9
Chain201D	KWIVWRWRFKR	[20]	1660	13	4	−1.1
FK-16Cys	FKRIVQRIKDFLRNLVC	[42]	2149	11	3	0.1
GL13K	GKIIKLKASLKLL	[19,39,41,48,53]	1429	11	4	0.7
HH27	GKWMKLLKKILK	[63]	1484	11	4	−0.3
HH39	GKWVKLLKKILK	[63]	1452	11	4	−0.1
hBD3-1	GINTLQKYYCRVRG	[30]	1670	10	3	−0.6
hBD3-2	GRCAVLSCLPKEQI	[30]	1516	8	1	0.4
hBD3-3	GKCSTRGRKCCRRKK	[30,61]	1767	11	8	−1.9
HHC36/tet127	KRWWKWWRR	[36,38,47,55,58,60]	1487	13	4	−2.8
KR-12	KRIVQRIKDFLR	[35,37]	1572	12	4	−0.7
KR-12/32	KIRVQRIKDFLR	[35]	1572	12	4	−0.7
KR-12/32-5911	KIRVRIKFR	[35]	1216	13	4	−0.6
KR-12-5911	KRIVRIKFR	[35]	1216	13	4	−0.6
Lam/LamLG3	KKGGGPPFLMLLKGSTRFC	[40,41]	2038	11	3	−0.1
LF1-11	GRRRRSVQWCA	[45]	1374	13	3	−1.4
LL37	LLGDFFRKSKEKIGKEFKRIVQRIKDFLRNLVPRTES	[35,55,62]	4493	11	5	−0.7
Mag-silk	GIGKFLHSAGKFGKAFVGEIMKS	[32]	2410	11	2	0.2
Mel4	KNKRKRRRRRRGGRRRR	[26,50]	2349	13	10	−3.8
Melimine	TLISWIKNKRKQRPRVSRRRRRRGGRRRR	[50,51]	3787	14	15	−2.3
Met11	NRIVQQRTSSR	[20]	1344	13	3	−1.6
ponericin G1	GWKDWAKKAGGWLKKKGPGMAKAALKAAMQ	[54]	3214	11	6	−0.7
RRP9W4N	RRPRPRPRPWWWW	[56]	1932	13	4	−2.5
SESB2V	[(RGRKVVRR)_2_K]_2_KK	[9]	4564	14	21	−2.0
Tet213	KRWWKWWRRC	[46,57]	1591	12	4	−2.2
β-peptide	(ACHC-β^3^hVal-β^3^hLys)_3_	[59]	1943	/	2	/
FN-silk	CTGRGDSPAC	[32]	966	6	0	−0.5
RGD	RGDS	[45]	433	7	0	−2.3
cHABP1	CMLPHHGAC	[47]	968	7	0	0.5
TBP-1	RKLPDAPGMHTW	[30,31]	1409	10	1	−1.0
TiBP/TiBP1	RPRENRGRERGL	[18,19]	1496	12	3	−2.6
TiBP2	SRPNGYGGSESS	[18]	1197	7	0	−1.6
TiBP1-Spacer-AMP	RPRENRGRERGLGGGLKLLKKLLKLLKKL	[18]	3342	12	9	−0.9
TiBP-spacer5-AMP	RPRENRGRERGLGSGGGLKLLKKLLKLLKKL	[5,29]	3485	12	9	−0.9
cHABP-1-Spacer-AMP	CMLPHHGACGGGKRWWKWWRR	[47]	2609	11	4	−1.0
DOPA-peptide	XXXXXKKKKKBXXXXXKKKKK **	[27]	3131	/	9	/
eAMP	RRRRRRGALAGRRRRRRGALAGEEEEEEE	[52]	3535	12	4	−2.2
TBP-1-hBD3-3	RKLPDAPGMHTWGGGGKCSTRGRKCCRRKK	[30,31]	3329	11	9	−1.4
TBP-1-RGDS-hBD3-1	RKLPDAPGMHTWGGGRGDSGGGGINTLQKYYCRVRG	[30]	3819	10	3	−0.9
TBP-1-RGDS-hBD3-2	RKLPDAPGMHTWGGGRGDSGGGGRCAVLSCLPKEQI	[30]	3665	9	2	−0.5
TBP-1-RGDS-hBD3-3	RKLPDAPGMHTWGGGRGDSGGGGKCSTRGRKCCRRKK	[30,31]	3915	11	9	−1.4
TiBP2-Spacer-AMP	SRPNGYGGSESSGGGLKLLKKLLKLLKKL	[18]	3042	10	6	−0.5
TiBP-AMPA	RPRENRGRERGLGSGGGKWKLWKKIEKWGQGIGAVLKWLTTW	[19]	4991	12	8	−1.0
TiBP-GL13K	RPRENRGRERGLGSGGGGGKKIKLKASLKLL	[19]	3218	12	7	−0.8
